# Supplementary material for: COVID-19 Lockdown: Impact on Oral Health-Related Behaviors and Practices of Portuguese and Spanish Children
Source: Int J Environ Res Public Health. 2022 Nov 30;19(23):16004. doi: 10.3390/ijerph192316004 (PMC9739203; doi:10.3390/ijerph192316004)
Supplement: Supplementary file 1 [file ijerph-19-16004-s001.zip › ijerph-2053471-supplementary.pdf]

This questionnaire is self-completed and is intended to assess the possible impact of COVID-19 confinement on the oral health of Portuguese and Spanish children. Answers must be based on ***the last period of confinement****, and each questionnaire* ***must concern only one child****. It is estimated that filling in does not take more than 5 minutes. All responses will be treated with criteria respecting confidentiality and anonymization.*

Thank you for your participation!

I agree to participate in this study, having been aware of its objectives by reading the informed consent (place hyperlink). I confirm that my participation as guardian of the child is voluntary and I understand that I can withdraw from filling in at any time, without any kind of prejudice.

a) Yes
b) No: **END OF QUESTIONNAIRE** (does not meet an inclusion criterion)

SOCIOECONOMIC DATA

About the child:

1. Age

____ years and ____ months

2. Gender

a. Female

b. Male

3. Country of residence

a. Spain

b. Portugal

c. Other: END OF QUESTIONNAIRE (does not meet an inclusion criterion)

4. Place of residence

a. Rural

b. Urban

5. Did the child have siblings in the household during the COVID-19 lockdown?

a. Yes

IF YES: How many of them were minors? Open question; quantitative variable

b. No

6. Did the child's lifestyle routine significantly changed because of COVID-19 confinement?

a. Yes

b. No

7. Did the child have online school activities during the COVID-19 lockdown?

a. Yes

b. No

8. Did the child experience any of the following emotional states or problems during COVID-19 lockdown? *(You can select one or more options)

a. Nervousness

b. Loneliness

c. Annoyance

d. Disinterest

e. Rage

f. Fear

g. Anxiety about going to public places

h. Other reason - specify ______

i. I don't know

j. I do not want to answer

About the respondent:

1. Did the person responding to this questionnaire live with the child during COVID-19 lockdown?

a. Yes

b. No: END OF QUESTIONNAIRE (does not meet an inclusion criterion).

2. What is your relationship with the child?

a. Dad

b. Mother

c. Grandfather or grandmother

d. Uncle or aunt

e. Brother or sister

f. Other relative, specify______

g. Other non-relative, specify______

3. Was the person who is responding to this questionnaire the child's primary caregiver during COVID-19 lockdown?

a. Yes

b. No

IF THE ANSWER WAS NO: Who was the child's primary caregiver during COVID-19 lockdown?

a. Dad

b. Mother

c. Grandfather or grandmother

d. Uncle or aunt

e. Brother or sister

f. Other relative, specify______

g. Other non-relative, specify______

About the child's family:

1. Education level of the child's primary caregiver?

a. No studies

b. Primary or secondary education

c. Bachelor's / Graduation

d. Master, Postgraduate or Doctorate

e. I don't know

f. I do not want to answer

2. What is the occupation of the child's mother?

a. Group I: Large businessmen, top managers in the public or private sector, university professors, senior military officers, liberal professions with higher education, senior political leaders

b. Group II: Medium-sized entrepreneurs, business managers, intermediate managers and technical staff in the public or private sector, armed forces officers, basic or secondary education teachers

c. Group III: Small businessmen, middle managers, medium farmers, sergeants or equivalent

d. Group IV: Small farmers and tenants, administrative technicians, semi-skilled workers, civil servants and members of the armed or militarized forces not yet mentioned

e. Group V: Agricultural wage earners, undifferentiated workers or professions not yet mentioned

f. I don't know

g. I do not want to answer

3. What is the occupation of the child's father?

a) Group I: Large businessmen, top managers in the public or private sector, university professors, senior military officers, liberal professions with higher education, senior political leaders

b) Group II: Medium-sized entrepreneurs, business managers, intermediate managers and technical staff in the public or private sector, armed forces officers, basic or secondary education teachers

c) Group III: Small businessmen, middle managers, medium farmers, sergeants or the like

d) Group IV: Small farmers and tenants, administrative technicians, semi-skilled workers, civil servants and members of the armed or militarized forces not yet mentioned

e) Group V: Agricultural wage earners, undifferentiated workers or professions not yet mentioned

f. I don't know

g. I do not want to answer

4. What was the employment status of the child's primary caregiver during COVID-19 confinement?

a. Worked outside the home part-time

b. Worked outside the home full-time

c. Worked from home part-time

d. Worked from home full time

e. Did not work and stayed at home

f. Other situation, such as: student, maternity or paternity leave

5. Did the child's family experienced significant income changes during COVID-19 lockdown?

a. Increase

b. Without changes

c. Slight reduction

d. Drastic reduction

e. Total loss

f. I don't know

g. I do not want to answer

6. What is the marital status of the child's parents?

a. Married

b. Divorced

c. Widower

d. In a relationship

e. Single

f. Other

g. I don't know

h. I do not want to answer

EATING HABITS

1. In general, did the child change his/her eating habits during COVID-19 confinement? *(You can select one or more options)

a. No changes

b. More snacks (intermediate meals)

c. Fewer snacks (in-between meals)

d. Higher intake of sugary and processed foods or beverages (e.g. soda, candy, and crackers)

e. Higher intake of healthy foods (e.g. fruits and vegetables)

f. I don't know

g. I do not want to answer

2. Excluding main meals (breakfast, lunch and dinner), on how many occasions a day did the child eat ~~food~~ during COVID-19 confinement?

a. One time

b. Twice

c. 3 times

d. 4 times

e. 5 or more times

f. I don't know

g. I do not want to answer

3. Please indicate the frequency of ingestion of each of the following foods (between main meals) during COVID-19 lockdown.

Snacks	Ingestion frequency						
	Less than once/ week	1- 3 times/ week	4-6 times/ week	Once a day	Twice a day	3 times/ day	4 or + times/ day
Dried fruits and/or fresh fruits							
Candy, chocolate							
Salty snacks (e.g. potato chips, salted popcorn, salted crackers)							
Cakes, sweet cookies, ice cream							
Sugar cereals, cereal bars							
Bread or sandwich							

4. Please indicate the frequency of ingestion of each of the following beverages during COVID-19 lockdown.

Beverages	Ingestion frequency						
	Less than once/ week	1- 3 times/ week	4-6 times/ week	Once a day	Twice a day	3 times/ day	4 or + times/ day
Homemade fruit juice							
Industrial fruit juice							
Soda							
Energetic drinks							
Sweetened/chocolate milk and/or sweetened yogurt							
Plain milk, dairy products							

ORAL HYGIENE HABITS

1. How often did the child toothbrush during COVID-19 confinement?

a. Not daily
b. Once a day
c. Twice a day
d. More than twice a day
e. I don't know
f. I do not want to answer

2. Was there any change in the child's toothbrushing frequency during COVID-19 confinement?

a. Unchanged
b. Increased
c. Decreased
d. I don't know
e. I do not want to answer

3. Did the child receive help/supervision during toothbrushing prior to COVID-19 lockdown?

a. Daily
b. Frequently
c. Occasionally
d. Never
e. I don't know
f. I do not want to answer

4. Did the child receive help/supervision during toothbrushing in COVID-19 confinement?

a. Daily
b. Frequently
c. Occasionally
d. Never
e. I don't know
f. I do not want to answer

5. How often did the child ~~children use dental~~ floss during COVID-19 confinement?

a. Not daily
b. Once a day
c. Twice a day
c. More than twice a day
d. I don't know
e. I do not want to answer

6. Was there ~~been~~ any change in the child's frequency of flossing ~~in children~~ during COVID-19 confinement?

a. Unchanged
b. Increased
c. Decreased
d. I don't know
e. I do not want to answer

7. Do you consider that the child's oral hygiene changed during COVID-19 confinement?

a. Unchanged
b. Got worse
c. Improved
d. I don't know
e. I do not want to answer

CHILD'S ORAL HEALTH STATUS AND ACCESS TO ORAL HEALTH CARE

1. How would you describe the child's oral health status during COVID-19 confinement compared to the previous period?

a. Unchanged
b. Got worse
c. Improved
d. I don't know
e. I do not want to answer

2. Was the child being followed up by the dentist before the COVID-19 confinement? *(You can select one or more options)

a. Yes, for dental caries treatment
b. Yes, for orthodontic treatment
c. Yes, for toothache
d. Yes, due to dental trauma
e. Yes, for another reason - specify _______
f. No
g. No, the child was never gone
h. I don't know
i. I do not want to answer

3. During COVID-19 lockdown, did the child have any of the following oral conditions that were not treated? *(You can select one or more options)

a. Dental caries
b. Gingival swelling
c. Gingival bleeding
d. Dental trauma
e. Loss or fracture of a dental restoration
f. Halitosis
g. None

h. I don't know

i. I do not want to answer

4. Did you detect any new caries lesions in the child's teeth during COVID-19 confinement?

a. Yes

b. No

c. I don't know

d. I do not want to answer

5. Did the child suffer any dental trauma during COVID-19 confinement?

a. Yes

b. No

c. I don't know

d. I do not want to answer

6. Did the child have a toothache during COVID-19 confinement?

a. Yes

b. No

c. I don't know

d I do not want to answer

7. Did the child attend any dental appointment during COVID-19 confinement?

A. Yes

IF YES: Why? *(You can select one or more options)

a. For a routine consultation

b. For a pre-scheduled dental treatment (e.g. orthodontic appointments)

c. For an urgent consultation for a toothache

d. For an urgent appointment due to an abscess

e. For an urgent appointment due to a dental trauma

f. For an urgent appointment due to tooth decay

g. For an urgent appointment due to a loss or fracture of a dental restoration

h. For another reason - specify ______

i. I don't know

j. I do not want to answer

B. No

IF THE ANSWER IS NO: Why? *(You can select one or more options)

a. Because there was no need

b. For fear of transmission of COVID-19 during dental treatment

c. Due to suspension of the activity of dental clinics

d. For financial reasons

e. Because dental treatment was not urgent

f. Because the child or her parents were infected with COVID-19

g. For another reason - specify ______

h. I don't know

i. I do not want to answer

8. Regarding the frequency of medical and dental consultations of the child during the confinement by COVID-19, compared to the previous period, I would consider that:

a. The child received less medical and dental follow-up during confinement

b. The child received more medical and dental care during confinement

c. Without changes

d. I don't know

e. I do not want to answer
